# Level of food consumption score and associated factors among pregnant women at SHEGAW MOTTA hospital, Northwest Ethiopia

**DOI:** 10.1186/s12889-021-10366-y

**Published:** 2021-02-06

**Authors:** Mehariw Birhan Ambaw, Getasew Shitaye, Mekuanint Taddele, Zewdie Aderaw

**Affiliations:** 1 Department of public health, Debre Markos University, College of Medicine and Health Science, Debre Markos, Ethiopia; 2 Biomedical Science Department, Bahir Dar university, College of Medicine and Health Sciences, Bahir Dar, Ethiopia

**Keywords:** Food consumption score, Factors, Pregnant women, East Gojjam, Northwest Ethiopia

## Abstract

**Background:**

Several studies conducted to access the status of household food insecurity in Ethiopia show that the nutrition problem is still highly prevalent especially in pregnant women and children. This study was conducted in 2018 main harvesting season with the principal objective to assess the level of food consumption score and associated factors among pregnant women attending antenatal service at Shegaw Motta Hospital.

**Methods:**

Institution based cross-sectional study was conducted among pregnant women attending antenatal care service at Shegaw Motta Hospital, East Gojjam Zone, Northwest Ethiopia. Primary data of 422 pregnant women were collected using interviewer administered structured questionnaire and a systematic random sampling technique was used to select study participants. The standardized World Food Program eight food groups English version questionnaire was translated to the local Amharic language and used along with the Ethiopian food composition table. The collected data were subjected to descriptive statistics and analyzed with SPSS software.

**Results:**

From the total of 422 pregnant women, 1.9% (95% CI: 0.7–3.3) of the respondents food consumption score were poor, 16.6% (95% CI: 13.0–20.4) were borderline and the remaining 81.5% (95% CI: 77.5–85.1) had acceptable food consumption score. Residence, being rural or urban [AOR = 4.594;95%CI: 1.871–11.283, *P* = 0.001], religion status, being an Orthodox [AOR = 0.073;95% CI: 0.021–0.254, *P* < 0.0001], were factors associated with food consumption score.

**Conclusions:**

Food consumption score among pregnant women seems to be highly unacceptable. Residence and religion were factors associated with food consumption score. Therefore, appropriate nutrition education should be given.

**Supplementary Information:**

The online version contains supplementary material available at 10.1186/s12889-021-10366-y.

## Background

Food insecurity is a situation that exists when people lack secure access to sufficient amounts of safe and nutritious food for normal growth and development and an active and healthy life [1]. Several studies conducted to access the status of household food insecurity in Ethiopia show that the nutrition problem is still highly prevalent especially in pregnant women and children [2]. Maternal under-nutrition diminishes woman productivity, causing bad results for herself, her family, her community, and the broader society. Maternal malnutrition is influenced not only by lack of adequate nutrition but also influenced by factors like socio demographic factors, nutritional knowledge of mother during pregnancies [3].

All human beings need a balanced amount of nutrients for proper functioning of the body system as nutrition is a fundamental pillar of human life, health and development [4]. The index of diversity and balanced food consumption is called Food Consumption Score (FCS) [5]. It is the core indicator of consumption hence it is a composite score based on dietary diversity, food frequency and relative nutritional importance of different food groups [5, 6].

Nutrition problem is still highly prevalent especially in pregnant women and children [2]. Maternal malnutrition is influenced by inadequate nutrition, socio demographic factors and poor nutritional knowledge of mother during pregnancies [3]. In the Ethiopian context, intra household food allocation is very sensitive, contributing to maternal malnutrition. Especially in the study area, evidence suggests that household food allocation is very maldistributed due to multiple factors that includes deep rooted culture, dietary habit and or attitude on women and children. Therefore, prevalence of food consumption score provides essential information on people’s current diets especially for pregnant women and children to enhance inputs into nutrition-sensitive program design [7, 8]. Hence, policy makers including food-based interventions in nutrition policies are dependent on studies on food insecurity and food consumption score to identify gaps and to act accordingly, FCS prevalence studies has a potential to give baseline information for improvements that will be done on intervention programs.

In Ethiopia, several food security studies show that many households, especially live in the rural areas, are food insecure. The problem is not only about food shortages but inadequate access of food [9, 10] and monotonous dietary pattern which is teff, wheat, corn, maize and sorghum are the most staple food items in the study area that are leads to poor food consumption [11]. Due to this poor food consumption both chronic and acute problems of food consumption are widespread and severe in both rural and urban areas of the country [12]. Even though, some study conducted about food insecurity which is a major indicator of food consumption score has given more emphasis to the rural area of the country do not identify situations at grass root level. Therefore, these partial assessments do not identify situations at grass root level rather hide the true food insecurity which is caused by poor variety of diet consumption. Furthermore, such studies do not look the underlying causes of food insecurity of household at the urban settings. The extent of food insecurity problem differs from place to place and in accordance to the social position and actual life conditions [7, 13]. The study on household capacities, vulnerabilities and food insecurity in Ethiopia [14] considering the food security over time also documented a significant difference in the level of food insecurity among rural, semi-urban and urban areas of the study population. In sort of seasonal food insecurity pattern, an empirical analysis of food insecurity in North Wello, Ethiopia evidenced that the area is highly food insecure along with other attitudinal variables being the potential influences of food insecurity [15]. Along with the findings of the food insecurity conditions in the region, studies also documented the influence of household food insecurity in the overall wellbeing and health of pregnant women. Accordingly, pregnant women who were from food insecure households seems to have lowered chances of attendance to antenatal care service [16] and pregnant women living in food insecure households were more likely to have mental distress than their counterparts [17]. Moreover, a similar study in Gambella town revealed that the prevalence of undernutrition among pregnant women was unacceptably high at which household food insecurity, low dietary diversity, and early marriage were reported as the predictors for undernutrition [18].

East Gojam zone have a huge potential of agricultural production. However, there is a documentation of very poor trend of food consumption [11] and also, considerable food insecurity for the past years in the area. A community-based comparative cross-sectional study among 4110 households in East and West Gojjam zones of Amhara Region conducted on level and determinants of food insecurity reported 55.3% total prevalence of food insecurity of which 59.2% of the East Gojjam and 51.3% of West Gojjam households were food insecure [19]. Similarly, a study in the same region conducted on maternal dietary diversity and micronutrient adequacy during pregnancy documented that the inadequate dietary diversity was prevalent in 55% among a total of 834 of pregnant woman [20]. Another recent study done [21] in selected districts of east Gojjam zone among 504 households founds that 31.15% were low food secure, and 43.25% were severely food insecure.

There are multiple factors that inhibit the availability component of food security. Among of all, participants’ religion and residence were found to be statistically significant with food consumption score during pregnancy. Fasting which is the most widely religious practice both in Christian and Muslim religion followers across the region should be highly considered in the food consumption score studies. It is also noteworthy that deep rooted culture on monotonous diet pattern, attitudes on dietary practice or conservative beliefs on food item preference and sociocultural barriers or attitudes are also understudied aspects that should be identified at grassroot level. Despite large number of studies conduct on the household food insecurity, almost there is no available study done on the level of food consumption score among pregnant women. On the other hand, the actual status of food consumption score and associated consumption constraints or factors like household food distribution, religion, socio-cultural barriers or attitudes and specifically dietary practice of pregnant women has not systematically studied in the area. This study, therefore, is conducted with the principal objective to assess the level of food consumption score and factors among pregnant women in the study area.

## Methods

### Study design and setting

Institution based cross-sectional study was conducted among pregnant women attending antenatal care service at Shegaw Motta Hospital. Shegaw Motta Hospital is located in East Gojjam Zone, Northwest Ethiopia, which is far from 372 Km from Addis Ababa to Northwest direction, 120 Km from Bahir Dar to south direction and 200 Km from Debre Markos. Regarding the dietary practice Teff, Wheat, Sorghum, Maize, Bean, Chick pea, Pea and other cereals and legumes are the commonest food sources whereas; fish and other seaweeds are not easy to access. The obstetric and gynecological care has started at the beginning of Shegaw Motta Hospital establishment 18 years ago. Currently the hospital is providing antenatal care for more than 9900 pregnant women per year and 1050 pregnant women were enrolled in 1 month and get the service with 17 health care providers that is 14 midwives, 1 gynecologist and 2 emergency surgery officers [11]. The study was conducted from February 23, 2018-April 3, 2018.

### Eligibility criteria

As inclusion and exclusion criteria, all pregnant women who were attended antenatal care service at Shegaw Motta Hospital and consumed only home prepared foods either at home or outside their home in the last 7 days during the data collection period were included. Whereas those who consumed any food item which was not prepared at home were excluded in this study.

### Sample size and sampling technique

The sample size was computed based on single population proportion formula assuming the prevalence (p) of food consumption score 50% (because of no previous study conducted in Ethiopia), 95% Confidence level (1.96), and 5% margin of error. Therefore, the final sample sizes for this study were 422. Power analysis was done for factor analysis but less than the first objective**.**

Systematic random sampling technique was employed to select study participants. According to Shegaw Motta Hospital ANC Report Sheet, in average a total of 1050 pregnant women attend ANC per month. Therefore, 422 study participants were selected by systematic random sampling technique. By taking the final sample size (*n* = 422), K was two. Thus, the study participants were selected every 2nd interval. To get the initial study participant, lottery method was used. Then, each study participant was selected every 2nd interval using systematic random sampling technique.

### Data collection tool and procedures

Data were collected using interviewer-administered structured questionnaire which was developed based on World Food Program standardized eight food frequency questions, Ethiopian Demographic and Health Survey and different literatures (Supplementary file 1). The collection of the data was executed during the main harvesting season in the study area and the tool contains socio-demographic characteristics, obstetric, attitude, knowledge, household assets and food consumption questions. Additionally, the data was collected in the study site when the study participants visited for their ANC service. There is standardized World Food Program eight food groups English version questionnaire [5] and this questionnaire was translated to the local Amharic language. To assess the food consumption score, the study participants were asked to recall all foods and beverages they have taken in the last 7 days prior to the interview. Using Ethiopian food composition Table [22], the local food items were categorized into eight food groups. The Ethiopian food composition table addresses all relevant local food items consumed across different regions of the country and gives standard weight value in line with the world food program eight food groups. For example, when participants responded that Injera was consumed, then the given weight value was recorded hence Injera is staple food prepared usually from teff though additional grain such as sorghum, maize, barley and wheat can be used occasionally. Condiments are generally eaten in a very small quantity often just for flavor, are not considered to have an important impact on overall diet and have zero weight value in the main food groups. In fact, there are also suggestions to design the questionnaire to address condiments during data collection; in this study weight cutoffs to distinguish between use of a food as a condiment and use as a main food are not used during data collection with the interviewees. But we generally isolated commonly used condiments and tried to manage in reference with the general standard questionnaire.

Another important aspect of the methodology of this research is applying the current food consumption score analysis at an individual level. To conduct this analysis with this new approach We relied on some reasons or assumptions. In the local context in the study area and the region at large, there is some evidence of a considerable feeding trend or habit in which women consumed their foods mostly after they feed their family. This condition for women may lead to unforeseen and underestimated shortage of food both in terms of availability and diversity. Therefore, we put this idea in a table for discussion with nutrition experts and after valuable recommendations and comments given, we decided that if the level of food consumption score is studied among pregnant women, it could provide a relevant and most informative results both to the study subjects and the household. Here, even though the appropriateness of the method was not fully validated elsewhere, this research is conducted with the intension to provide new insights and relevant information to stakeholders.

All consumption frequency of foods in the same group were summed and multiplied with value of each food group by its weight. Then the weight of food groups score was summed to obtain food composition score to determine the status of pregnant women food consumption score based on the following result: 0–21 score was poor; 21.5–35 score was borderline and **>** 35 score was acceptable. In general, if food consumption score is < 35 it indicates the presence of household food insecurity [5].

Two professional midwives as data collectors and one Gynecologist as a supervisor were recruited in the study. Prior to data collection, data collectors and supervisor were trained by a nutritionist on how to they interview the participants and also enable them to gain basic concepts on FCS. The supervisor and principal investigator were followed the data collection technique. All the data collectors and the supervisor had regular meeting with the principal investigator at the end of each day of data collection period.

### Data management and analysis

The collected data was coded and entered into Epi-Data Version 3.1 and cleaned. The cleaned data set was exported to SPSS Version 23.0 software for analysis. Study participants food consumption score (poor, borderline and accepted), socio-demographic characteristics and other independent variables were presented using relevant descriptive statistics. Wealth index factor analysis was computed by the application of principal component analysis (PCA) as described in by Lisa H et al., 2017. Initially household asset data were prepared for analysis. Before the PCA, using frequency, important variables that can discriminate households were selected to reduce number of variables. The binary variables were coded to 0 and 1. After data preparation, variables were standardized to change variables in to the same scale for comparison, the variables have mean of zero and standard deviation of 1. Among A total of 17 variables that were considered for wealth index construction, 12 variables were dropped as their communality scores were less than 50% and the rest five variables, including number of cattle in the household, number of rooms in house, having commercial bank account, presence of agricultural land including irrigation and type of latrine to the household were considered for wealth index construction.

Univariate analysis was done at 25% level of significance to screen out potentially significant independent variables. Multiple Logistic Regression was performed to see the association between the dependent variable and independent variables. The adequacy of the final model was checked using Hosmer and Lemeshow goodness of fit test, and an assumption of Binary Logistic Regression such as no multi-co linearity was checked. For Binary Logistic Regression, 95% confidence interval was calculated and variables with *p*-value < 0.05 were considered as statistically significant with the outcome variable (food consumption score).

## Results

### Demographic characteristics

A total of 422 study participants were included in the study making a response rate of 100%. More than two third (73.2%) of study participants live in urban area. The vast majority of the pregnant women were married (99.1%) and Orthodox Christian in religion (71.3%). The mean (±Standard deviation) age of the study participants was 27.64(±5.426) years, about 58.1% were in the age range 25–34 years. Among those study participants, 25% of pregnant women had attended diploma and above educational status and 37.6% of pregnant women were merchants. Regarding wealth status of pregnant women 114 (27%) of the study participants were poorer, whereas 107 participants (25.4%) were rich. Other characteristics of the study population are given in Table 1.

**Table 1:** Socio-demographic characteristics of pregnant women attending ANC service at. Shegaw Motta Hospital, Northwest Ethiopia, 2018 (*n* = 422)

Variables	Overall n (%)
**Age in years**	
18–24	124 (29.4)
25–34	245 (58.1)
35–49	53 (12.6)
**Marital status**	
Married	418 (99.1)
Unmarried	4 (0.9)
**Residence**	
Urban	309 (73.2)
Rural	113 (26.8)
**Religion**	
Orthodox	301 (71.3)
Others	121 (28.7)
**Educational status**	
Cannot read and write	88 (20.9)
Can read and write	39 (9.2)
Grade 1–8	107 (25.4)
Grade 9–12	81 (19.2)
Diploma and above	107 (25.4)
**Occupation**	
Merchant	157 (37.6)
Governmental	135 (32.3)
Farmer	85 (20.3)
Others	41 (9.8)
**Husband education**	
Cannot read and write	53 (12.7)
Can read and write	52 (12.4)
Grade1–8	72 (17.2)
Grade9–12	96 (23)
Diploma and above	145 (34.7)
**Husband occupation**	
Government employee	135 (32.3)
Merchant	157 (36.7)
Farmer	85 (20.3)
Daily laborer	2 (0.5)
others	39 (9.3)
**Wealth index**	
Richest	75 (17.8)
Rich	107 (25.4)
Middle	61 (14.5)
Poorer	114 (27)
poorest	65 (15.4)

### Food consumption score

The current magnitude of unacceptable food consumption score (poor or borderline) in this study was found to be 18.5%. Among the study participants, the majority (81.5%) of participants food consumption score was acceptable.

### Factors associated with food consumption score

Nearly 51.7% of the study subjects were at the third trimester by gestational age while about 66.8% of participants were multigravida. 92.4% and greater than three fourth (91.7%) of study subjects did not have any history of still birth and abortion in their reproductive life respectively (Table 2).

**Table 2:** Univariate and Multivariable analysis for factors associated with food consumption score among pregnant women attending ANC service at Shegaw Motta Hospital, Northwest Ethiopia, 2018 (*n* = 422)

Variables	Food Consumption Score				
	Unacceptable	Acceptable	COR(95%CI)	AOR(95%CI)	***P***-Value
**Age**					
18–24	42	203	1.00	1.00^b^	
25–34	17	36	2.282(0.618, 9.96)	1.451(0.705, 9.86)	0.312
35–49	22	102	1.042(0.591, 1.840)	1.025(0.333, 3.159)	0.966
**Residence**					
Urban	37	272	1.00	1.00^b^	
Rural	44	69	4.688(2.813, 7.813)	**4.594(1.871, 11.283)**	0.001**
**Religion**					
Orthodox	78	223	1.00	1.00^b^	
Others^a^	3	118	0.073(0.022, 0.235)	**0.073(0.021, 0.254)**	< 0.0001**
**Education**					
Not read	26	62	0.298(0.130, 0.684)	0.748(0.158, 3.548)	0.715
Read	12	27	0.281(0.107, 0.743)	0.208(0.055, 0.781)	0.07
G1–8	16	91	0.711(0.297, 1.702)	0.789(0.277, 2.245)	0.657
G9–12	18	89	0.618(0.262, 1.458)	0.559(0.166, 1.884)	0.348
> =Diploma	9	72	1.00	1.00^b^	
**Occupation**					
Governmental	16	77	1.00	1.00^b^	
Merchant	14	95	3.391(1.670, 6.882)	1.932(0.365, 10.225)	0.439
Farmer	2	10	4.781(2.315, 9.874)	0.924(0.200, 4.257)	0.919
Daily labor	18	115	3.523(0.721, 17.21)	0.652(0.042, 10.020)	0.759
others	31	44	4.501(2.288, 8.856)	1.877(0.503, 6.997)	0.348
**Knowledge**					
Knowledge able	51	147	1.00	1.00^b^	
Not knowledgeable	30	196	2.244(1.362, 3.696)	1.683(0.861, 3.288)	0.128
**Wealth Index**					
Richest	18	47	1.00	1.00^b^	
Rich	20	87	1.666(0.418, 1.880)	2.244(0.819, 6.142)	0.116
Middle	13	48	1.414(0.724, 3.008)	1.463(0.622, 3.440)	0.383
Poorer	11	36	1.253(0.561, 2.799)	1.433(0.513, 4.002)	0.492
Poorest	7	56	3.064(1.412, 7.147)	3.258(1.221, 8.698)	0.18
**Gestational Age**					
< =12 weeks	3	10	0.992(0.263, 3.745)	1.355(0.215, 8.518)	0.746
12-28 weeks	28	163	1.733(1.040, 2.886)	1.681(0.925, 3.054)	0.088
> =29 weeks	50	168	1.00	1.00^b^	
**Still birth**					
Yes	9	23	0.579(0.257, 1.303)	1.722(0.619, 4.794)	0.298
No	72	318	1.00	1	
**Abortion**					
Yes	12	23	0.416(0.197, 0.876)	0.461(0.176, 1.211)	0.116
No	69	318	1.00	1.00^b^	

Note: *COR* Crude odds ratio, *AOR* Adjusted odds ratio, *CI* Confidence interval

** Statistically significant

Others^a^ include Muslim, Protestant and Catholic religion followers

1.00^b^ reference category

In the univariate analysis, twelve independent variables, namely, participants’ age, education status, occupation, residence, religion, knowledge, history of abortion, history of still birth, husband’s employment and occupation status, gestational age, and wealth status were found with a *P*- value of < 0.25 and fitted to the multivariable logistic regression model at 5% level of significance. However, in the adjusted analysis, participants’ residence and religion were significantly and independently associated with poor/borderline food consumption score.

Those pregnant women who were Muslim, Protestant and Catholic followers referred as ‘others’ in the table were nearly 92.7% less likely to have unacceptable food consumption score as compared to those who were Orthodox Christian followers. The odds of poor/borderline food consumption score were decreased by 2.7% (AOR =0.073, 95% CI: 0.021, 0.254, *P* < 0.001) in pregnant women whose religion are Muslim, Catholic, Protestant and others as compared to those pregnant women who were Orthodox.

Regarding participants residence; living in the rural area were 4.594 times more likely to have poor/borderline food consumption score as compared to those who were living in the urban area (AOR = 4.594, 95% CI: 1.871, 11.283). Details of factors associated with food consumption score among pregnant women are presented in Table 2.

## Discussion

Nutrition is intimately and inextricably involved in all aspects of human growth and development. Despite the needs of a balanced amount of nutrients for proper functioning of the body system, chronic energy deficiency is caused by eating too little or having an unbalanced diet that lacks adequate nutrients. Pregnant women are especially vulnerable to chronic energy deficiency due to low dietary intake, inequitable distribution of food within the household, improper food storage and preparation, dietary taboos, infectious diseases, and inadequate care practices [23, 24]. It is well known that chronic energy deficiency leads to low productivity among reproductive ages and is related to morbidity and mortality. In addition, chronic under nutrition among women is a major risk factor for adverse birth outcomes [25].

The global nutrition and health agenda is concerned and designed to support the community’s efforts [26] in the global nutrition targets for 2025. Prominently, the issue of household food security has also received increased attention in the last couple of decades worldwide particularly due to worsening economic conditions. Since then many literatures that could potentially benefit the improvements in the global health have been produced on food insecurity studies in different settings.

Another very concerning and current topic of the scientific world in nutrition is the food consumption score which is a catalyst towards improved nutrition sensitive and specific programs by identifying nutrient inadequacies in households. It has been studied and documented that socio-demographic, obstetric, knowledge, attitude and household assets related characteristics are some of the key factors associated with the level of food consumption score among pregnant women [4, 27–31]. Notably, unacceptable food consumption score is the major public health problem [32] and therefore strengthening nutrition intervention is unduly essential [12].

The present study addressed two important issues. One is the level of food consumption score among pregnant women attending antenatal care service at Shegaw Motta Hospital, North West Ethiopia and the other is its associated factors with level of food consumption score. Importantly, the current magnitude of unacceptable food consumption score (poor or borderline) in this study was found to be 18.5 and 81.5% of participants having acceptable food consumption score. The study showed significantly higher magnitude of unacceptable food consumption score as compared to previous studies conducted in Ethiopia; Amhara (11%), Tigray (16%), Afar (6%), and Somali (17%) [33] but, still the figure is unacceptably high. This could be poor utilization of food, poor dietary diversity practice and consumption of cereal with pulses is common in these areas. Another explanation could be decreasing in public awareness towards proper food utilization from time to time and ecological difference between two study settings could describe this discrepancy. Besides, availability issue, cultural issue and seasonal variation may explain the difference. Additionally, the figure from this finding supports the previous reports from cross-sectional study done in Addis Ababa [34]. However, another survey conducted in Afar region among refugee population in 2011 [35], the prevalence of food consumption score for poor, underline and acceptable was 1.104, 4.6 and 94.28 respectively which is least comparable to our findings. Interestingly enough, the reports from this study is in line with 9% poor FCS and 21% a borderline in rural zone as reported from analysis of the food consumption score in Uganda, but lower than 39.13% poor, 5.4% borderline and 40.5% acceptable food consumption score prevalence in Burundi [33].

The figure (18.5%) is considerably higher than reports from some developing countries. From previous national food consumption score vulnerability assessment and mapping it has been reported that the level of food consumption score in Ethiopia was 74% [33], which is far lower than recommended by WFP which is 90% [35] but this study finding is 81.5%; this discrepancy may be due to the residence of the respondents, poor food consumption utilization and mal distribution of food. Additionally, it could be, participants who were enrolled in this study were small in number which results in difficulty of generalizability, gestational age, wealth status, geographical difference and methodological variation.

The other significant finding of this research is the association of key factors with the level of food consumption score among the study participants. Among considerably large number of factors analyzed the current study came up with the evidence of significant association between respondents’ residence and religion with food consumption score during pregnancy. This is supported by a study from Nigeria, Uganda and Ethiopia in which to be rural is responsible factor for the unacceptable food consumption score [35–37]. High prevalence of under-nutrition and poor dietary practice among pregnant woman in rural communities of Illu Aba Bor Zone, Southwest Ethiopia [38] may also further support the finding of this research. This consumption score variation across place of the residences could be explained by the different level of awareness among two groups of population and cost of accessing better nutrition like diary product and other diets which are enrich with micronutrients. Differently, from our finding other studies about determinants of food consumption, economic and demographic factors such as household sizes, dependency ratios, education, employment status, gender and women nutrition information were reported as the most significant factors [31, 34].

In this study those pregnant women who were Muslim, Protestant and Catholic followers were nearly 92.7% less likely to have unacceptable food consumption score as compared to those who were Orthodox Christian followers. The difference might be because of fasting and food preference among the study subjects and in fact this result is supported by a review on religious recommendations on diet and life style all over the world and Ethiopian [39, 40]. The association of food consumption score with religion in this study is also strengthened by a study in the eastern Cape, South Africa that documented food taboos and cultural beliefs influences on food choice and dietary preferences of pregnant women [41]. Furthermore, another study on factors influencing dietary patterns during pregnancy in a culturally diverse society further supported the reports from this study [42].

## Conclusion

The magnitude of unacceptable food consumption score among pregnant women attending ANC at Shegaw Motta Hospital seems to be high. Participants’ religion and residence were found to be statistically significant with food consumption score during pregnancy. Therefore, this study suggests that more specific nutrition programmes should be developed to improve the nutritional status of pregnant women. It also highlights the need to strengthen the religion-based nutrition education regarding nutritional knowledge of mothers including the necessity of feeding diverse and balanced food items. Moreover, given special attention for pregnant women especially for those who are Orthodox Christian followers and living in rural areas, health professionals, the community and other stakeholders should be intensively involved by advocating the current novel strategies (1000+ days).

In culturally diverse and religion rich people the nature of their religion like fasting practices might be either positive or negative force for the nutritional status of individuals. It should be noted that religious recommendations or educations are not to interfere the central dogma of the followers, not to change the religious practices but finding other options to overcome the problems and nutrition education creating awareness. We believe that education can change the attitude of the society and this works in other sectors. Hence, nutrition experts should advocate, health extension workers should educate the local society, woreda and zone stakeholders must be part of the solution and guidebooks considering religious practices/views should be available at many levels in the country. Though, food security and FCS seems two sides of one coin, improvements in food security does not achieved solely by such recommendations. Further researches should be done to explore scientific classification of food consumption score by ordinal logistic regression as suggested in standard food consumption score guideline.

### Limitations of the study

This study has some limitations. We did not validate the current standard food consumption score methodology for its use at individual level. It is not done using ordinal logistic regression. Institution based cross-sectional design was applied and it would be good to consider cohort study at community level for further researches.

## Supplementary Information

**Additional file 1: Supplementary file 1** Questionnaire, English language version”.

## Data Availability

The datasets used and/or analyzed during the current study are available from the corresponding author on reasonable request.

## Abbreviations

FCS: Food Consumption Score
PCA: Principle Component Analysis
