# Supplementary material for: Level of food consumption score and associated factors among pregnant women at SHEGAW MOTTA hospital, Northwest Ethiopia
Source: BMC Public Health. 2021 Feb 6;21:311. doi: 10.1186/s12889-021-10366-y (PMC7866766; doi:10.1186/s12889-021-10366-y)
Supplement: Supplementary file 1 — Additional file 1: Supplementary file 1 Questionnaire, English language version”. [file 12889_2021_10366_MOESM1_ESM.docx]

# APPENDEX

## Information Sheet

A cross sectional study questionnaire to assess food consumption score and associated factors among pregnant women at Shegaw Motta hospital, Northwest Ethiopia, 2018.

My name is…………………. I am collecting data on the behalf of Mr. Mehariw Birhan. He is final year Public Health Nutrition student at Debre Markos, University College of health Sciences.

He is currently conducting a research as partial fulfillment for the requirement of Master of Public Health in Nutrition at Markos University on the topic of “food consumption score and associated factors among pregnant women at Shegaw Motta hospital” .You are selected to be one of the participants in the study and you are kindly requested to give necessary information. If you agree to give the information, you will be requested to answer for questionnaire including socio-demography. In this form your name will not be described and the information you give are kept confidential. If you do not want to answer all or some of the questions, you do have the right to do so. However your willingness to answer all of the questions is very important to identify the prevalence of food consumption score and associated factors and to design nutrition interventions.

Would you participate in responding to the questions in this questionnaire? Yes No

## Consent Form

If you have any questions you may ask me now or later, even after the study has started. If you wish to ask questions later, you may contact any of the following individuals:

1. Mehariw Birhan

Cell phone: 0913725100

Mail: meharibirhan@gmail.com

You can ask me any more questions about any part of the research study, if you wish to. Do you have any questions?

The information is reading to me. I have the opportunity to ask questions about it and any questions that I have asked have been answered to my satisfaction. I consent voluntarily to participate as a participant in this research.

ID No. of Participant__________________

Signature of Participant ___________________

Date ___________________________ Day/month/year

**Consent for those who are unable to read and write**

I have witnessed the accurate reading of the consent form to the potential participant, and the individual has an opportunity to ask questions. I confirm that the individual has given consent freely.

Name of witness_____________________ AND Thumb print of participant

Signature of witness ______________________

Date ________________________ Day/month/year

**Statement by the data collectors**

I have accurately readout the information sheet to the potential participant and to the best of my ability make sure that the participant understands that the following will be done:

1. Interviewing questionnaires

I confirm that the participant will be given an opportunity to ask questions about the study, and all the questions asked by the participant will be answered correctly and to the best of my ability. I confirm that the individual will not be forced into giving consent, and the consent will be given freely and voluntarily.

Name of a person taking the consent________________________

Signature of a person taking the consent______________________

Date ___________________________ Day/month/year

**To be filled by data collectors and supervisors**

| Name of data collectors& supervisors |  | Signature |
| --- | --- | --- |
| Data collector name | ___________________ | ___________________ |
| Supervisor name | ____________________ | ___________________ |
| Data collection date | ___________________ |  |

## Data Collection Tool

### English Version Questionnaire

**Part one: Socio-demographic Characteristics of the Respondents**

Code No.___________________________

| **S.No.** | **Questions** | **Alternatives** | **Skip** |
| --- | --- | --- | --- |
| 101 | Age | --------------years |  |
| 102 | Place of Residence | 1. Urban 2. Rural |  |
| 103 | Religion | 1. Orthodox 2. Muslim 3. Protestant 4. Catholic 5. Others specify______ |  |
| 104 | Ethnicity | 1. Amhara 2. Oromo 3. Tigre 4. Others (specify)______ |  |
| 105 | Educational status | 1. Cannot read and write 2. Can read and write 3. Grade1-8 4. Grade 9-12 5. Diploma and above |  |
| 106 | Occupation | 1. Government employee 2. Merchant 3. Farmer 4. House wife 5. Daily laborer 6. Student 7. Other specify |  |
| 107 | Marital status | 1. Married 2. Single 3. Divorced 4. Widowed | If your answer is 2,3&4Skip to Q110 |
| 108 | If married what is your husband educational status? | 1. Cannot read and write 2. Can read and write 3. Grade1-8 4. Grade 9-12 5. Diploma and above |  |
| 109 | Husband occupation | 1. Government employee 2. Merchant 3. Farmer 4. Daily laborer 5. Student 6. Others (specify)____ |  |

**Part two: World Food Program (WFP) Standardize Food Frequency Questionnaires**

| **S.No.** | **Food items** | **Food groups** | **Frequency** |
| --- | --- | --- | --- |
| 110 | Rice, wheat, maize, barley, maize porridge, sorghum, millet, pasta, bread and other cereals | Main staples |  |
|  | Potato and sweet potatoes |  |  |
| 111 | Beans, chickpea, Peas, groundnuts, soya beans, | Pulses |  |
| 112 | Cabbage, chills, pumpkin, onion, tomato | Vegetables |  |
| 113 | Banana, orange, apple, mango, papaya, lemon | Fruit |  |
| 114 | Beef, goat, poultry, pork, eggs and fish | Meat and fish |  |
| 115 | Milk yogurt and other diary | Milk |  |
| 116 | Oils, fats and butter | Oil |  |
| 117 | Sugar and sugar products | Sugar |  |

**Part three: Obstetric questionnaires**

| **S.No.** | **Questions** | **Responses** | **Skip** |
| --- | --- | --- | --- |
| 118 | Gestational age | ___________weeks |  |
| 119 | Gravidity (how many times that you have been pregnant?) | __________in number |  |
| 120 | Parity (how many times that you give birth?) | __________in number |  |
| 121 | Number of ANC visit including the current one | ___________in number |  |
| 122 | Is there any history of still birth? | 1. Yes 2. No |  |
| 123 | If yes for Q No.122 how many times? | __________ in number |  |
| 124 | Is there any history of abortion | 1. Yes 2. No |  |
| 125 | If yes for Q No.124 how many times? | ___________ in number |  |

**Part four: Wealth index related characteristics**

| **S.No.** | **Questions** | **Alternatives** |
| --- | --- | --- |
| 126 | Ownership of the house | 1. Private 2. Rented from individual 3. Others (specify)________ |
| 127 | How many rooms are there in your home? | __________in number |
| 128 | What is the main material of the dwelling floor? | 1. Earth / Sand  2. Cement  3. ceramic  4. Bamboo  5. Carpet  6. Others (specify)_________ |
| 129 | What is the main material of the roof? | 1. Iron corrugated sheet  2. Wood  3. Thatch  4. Bamboo  5. Others (specify)_________ |
| 130 | What is the main material of the exterior walls? | 1. Stone with mud 2. Wood with mud 3. Stone with cement 4. Others (specify)_________ |
| 131 | What type of fuel mainly used for household cooking? | 1. Electricity 2. Charcoal 3. Wood 4. Animal dung 5. Others (specify)___ |
| 132 | Is the cooking usually done in the house, in a separate building, or outdoors? | 1. In a separate room used as kitchen  2. Elsewhere in the house  3. In a separate building  4. Other *(*specify*)________* |
| 133 | How many hector of agricultural land including irrigation land do you have? | ______________ |
| 134 | Annual total agricultural products(includes all items) | ___________kuintal |
| 135 | Does your household have   1. Electricity? 2. A Radio? 3. A Television? 4. A Non-mobile telephone? 5. A Refrigerator? 6. Table? 7. Chair? 8. A bed with cotton/spring mattress | \| Yes \| No \| \| --- \| --- \| \| 1 \| 2 \| \| 1 \| 2 \| \| 1 \| 2 \| \| 1 \| 2 \| \| 1 \| 2 \| \| 1 \| 2 \| \| 1 \| 2 \| \| 1 \| 2 \| |
| 136 | Does any member of your household own   1. A watch? 2. A mobile phone? 3. A bicycle? 4. A Bajaj? 5. Animal drawn cart? 6. Car? | \| Yes \| No \| \| --- \| --- \| \| 1 \| 2 \| \| 1 \| 2 \| \| 1 \| 2 \| \| 1 \| 2 \| \| 1 \| 2 \| \| 1 \| 2 \| |
| 137 | Does this household own any livestock, herds, other farm animals, or poultry? | 1. Yes 2. No |
| 138 | How many of the following animals do the household have? (if Q137 answer is 2 skip to Q139) |  |
|  | 1. Cattle, milk cows, bulls? | _________in number |
|  | 1. Horses, Donkeys, or mules? | _________in number |
|  | 1. Goats? | _________in number |
|  | 1. Sheep? | _________in number |
|  | 1. Chickens? | _________in number |
|  | 1. Beehives? | _________in number |
| 139 | Do you have Bank account or Amhara credit and saving institution? | 1. Yes  2. No |
| 140 | If yes Q139, how much money do you have in the bank or Amhara credit and saving institution? | ____________ETB |
| 141 | Where do you access drinking water | 1. Tape water 2. Protected spring 3. Unprotected spring/river |
| 142 | Do you have a latrine? | 1. Yes 2. No |
| 143 | If yes Q142, what type of latrine facility do you have? | 1. Flush to piped sewer system 2. Flush to septic tank 3. Ventilated improved pit (VIP) latrine 4. Pit latrine without VIP a slab 5. Open field |

**Part five: Maternal Attitude towards consumption of variety of diet**

| **S.No.** | **Questions** | **Strongly agree** | **Agree** | **Neutral** | **Disagree** | **Strongly Disagree** |
| --- | --- | --- | --- | --- | --- | --- |
| 144 | Consumption of meat and vegetable has different values on health. |  |  |  |  |  |
| 145 | Consumption of cereals, legumes, meat and milk food at the same time has a harmful effect on health. |  |  |  |  |  |
| 146 | Consumption of food on right quantity and time will support human growth and ensure health. |  |  |  |  |  |
| 147 | Consumption of vegetables and fruits can prevent from different diseases. |  |  |  |  |  |

**Part six: Maternal knowledge about food consumption during pregnancy**

| 148 | Have you heard about food consumption system?(if no skip to Q150) | 1. Yes 2. No |
| --- | --- | --- |
| 149 | If your answer is yes for 148, what is the source of information? | 1. Radio 2. TV 3. Printed materials 4. Relatives/friends 5. Health workers 6. Others (specify) ______ |
| 150 | What is the importance of following correct food consumption system during pregnancy? (**Multiple responses are possible)** | 1. Prevent Mental retardation 2. Prevents of anemia 3. Growth and development 4. Others(specify) ______ 5. I don’t know |
| 151 | What is the richest source of protein, carbohydrate, iron? (**Multiple responses are possible)** | 1. Egg 2. Meat 3. Milk and milk product 4. Fruit and vegetable 5. Fish 6. Others (specify__________ 7. I don’t know |
| 152 | Do you know that food consumption of pregnancy different from un pregnant one? | 1. Yes 2. No |
| 153 | Can you list any problems on pregnant women and fetus due to inadequate food consumption? (**Multiple responses are possible)** | 1. Mental retardation 2. anemia 3. growth failure 4. Abortion 5. Child mortality 6. Others(specify)_______ 7. I don’t know |
| 154 | Is there any guideline in Ethiopia that promotes variety of diet consumption during pregnancy? | 1. Yes 2. No |
